# MicroRNA-124-loaded nanoparticles increase survival and neuronal differentiation of neural stem cells *in vitro* but do not contribute to stroke outcome *in vivo*

**DOI:** 10.1371/journal.pone.0193609

**Published:** 2018-03-01

**Authors:** Cláudia Saraiva, Daniela Talhada, Akhilesh Rai, Raquel Ferreira, Lino Ferreira, Liliana Bernardino, Karsten Ruscher

**Affiliations:** 1 Health Sciences Research Centre, Faculty of Health Sciences, University of Beira Interior, Covilhã, Portugal; 2 Laboratory for Experimental Brain Research, Division of Neurosurgery, Department of Clinical Sciences, Wallenberg Neuroscience Center, Lund University, Lund, Sweden; 3 Departamento de Química, Faculdade de Ciências e Tecnologia da, Universidade Nova de Lisboa, Caparica, Portugal; 4 CNC - Center for Neuroscience and Cell Biology, Coimbra, Portugal; 5 Faculty of Medicine, University of Coimbra (IIIUC), Coimbra, Portugal; Fraunhofer Research Institution of Marine Biotechnology, GERMANY

## Abstract

There is a high quest for novel therapeutic strategies to enhance recovery after stroke. MicroRNA-124 (miR-124) has been described as neuroprotective and anti-inflammatory molecule. Moreover, miR-124 is a well described enhancer of adult neurogenesis that could offer potentially beneficial effects. Herein, we used miR-124-loaded nanoparticles (miR-124 NPs) to evaluate their therapeutic potential in an *in vitro* and *in vivo* model of stroke. For that, neuroprotective and neurogenic responses were assessed in an *in vitro* model of stroke. Here, we found that miR-124 NPs decreased cell death and improved neuronal differentiation of subventricular zone (SVZ) neural stem cell cultures after oxygen and glucose deprivation. In contrast, intravenous injection of miR-124 NPs immediately after permanent focal ischemia induced by photothrombosis (PT) did not provide a better neurological outcome. In addition, treatment did not affect the number of 5-bromo-2'-deoxyuridine (BrdU)- and doublecortin/BrdU- positive cells in the SVZ at the study endpoint of 14 days after PT. Likewise, the ischemic insult did not affect the numbers of neuronal progenitors in the SVZ. However, in PT mice miR-124 NPs were able to specifically augment interleukin-6 levels at day 2 post-stroke. Furthermore, we also showed that NPs reached the brain parenchyma and were internalized by brain resident cells. Although, promising *in vitro* data could not be verified *in vivo* as miR-124 NPs treatment did not improve functional outcome nor presented beneficial actions on neurogenesis or post-stroke inflammation, we showed that our NP formulation can be a safe alternative for drug delivery into the brain.

## 1. Introduction

After stroke, the adult brain attempts to compensate lost function by reorganizing itself, an action that involves multiple interconnected mechanisms such as cell genesis, astrogliosis, inflammation and neuronal plasticity. The proliferation and differentiation of cells derived from neural stem cells (NSCs) may replace lost neurons and thereby contribute to improve functional deficits [1–3]. In addition, inflammatory cascades, either detrimental or beneficial, significantly contribute to acute tissue demise. However, an increased activation of immune cells as well as inflammatory molecules can be observed weeks after the insult and may contribute to restoration of brain function [4]. Interestingly, therapeutic experimental approaches targeting detrimental inflammatory cascades have been translated into clinical trials aiming at improving neurological outcome of stroke patients, reviewed at Lakhan et al., 2009 and Simats et al., 2016 [5,6].

MicroRNAs (miR) are small endogenous, non-coding RNAs able to regulate hundreds of genes at the post-transcriptional level by inhibiting mRNA translation or inducing mRNA degradation [7]. Previous reports showed that miR-124 levels were decreased in neural progenitor cells of the subventricular zone (SVZ) and in the ischemic core [8,9], but seemed to be elevated in the plasma of rodents subjected to permanent occlusion of the middle cerebral artery (MCAO) [10,11]. In stroke patients, downregulation of plasma levels of miR-124 within the first 24 h was negatively associated with infarct size [12]. In contrast, another study showed increased plasma levels of miR-124 and those were correlated with higher mortality during the first 3 months after stroke and a worse outcome based on post-stroke modified Rankin Score (mRS) [13]. In stroke models, overexpression of miR-124 prior to stroke decreased infarct volume, reduced microglial activation and improved neurogenesis via ubiquitin-specific protease (Usp)14-dependent REST degradation [14,15]. In addition to protective effects, injection of liposomated miR-124 into the striatum of mice two days after transient MCAO promoted an anti-inflammatory state (M2 state) of microglia/macrophages and conversely reduced their pro-inflammatory state (M1 state) correlated with a better functional outcome during the first week after stroke onset [16,17]. In contrast, others have demonstrated that downregulation of miR-124 resulted in lower infarct volumes while no changes in terms of infarct volumes have been observed after overexpression of miR-124 [18,19].

MicroRNAs are small molecules with short half-life and poor stability. To overcome this issue we have developed ~210 nm-size polymeric NPs with a fluorine compound that can be tracked by fluorine (^19^F) magnetic resonance imaging (MRI) [20]. This system has already proven efficacy in miR delivery into cells both *in vitro* and *in vivo*. For example, we demonstrated, using this formulation, that intracerebroventricular delivery of miR-124-loaded NPs (miR-124 NPs) promotes SVZ neurogenesis in mice both in physiological conditions and after 6-hydroxydopamine lesions. We have also shown that administration of miR-124 NPs increased the number of new neurons in the lesioned area associated with amelioration of Parkinson’s disease-related motor deficits [21].

The present study has been conducted to evaluate if exposure of miR-124 NPs affects survival and differentiation of SVZ derived NSCs after exposure to oxygen and glucose deprivation (OGD). Positive results from these experiments prompted us to hypothesize that systemic administration of miR-124 affects stroke outcome measures, namely neurological functions during the first 14 days after photothrombotic stroke (PT). Moreover, we sought to identify the role of miR-124 on post-stroke inflammatory response and neurogenesis.

## 2. Materials and methods

### 2.1 Synthesis of poly(lactic-co-glycolic acid) (PLGA) NPs

PLGA NPs were prepared as described by Gomes and colleagues [20]. Briefly, PLGA (Resomers 502 H; 50:50 lactic acid/glycolic acid; Boehringer Ingelheim Lda, Ingelheim, Germany) was covalently conjugated to fluoresceinamine (Sigma-Aldrich Co. LLC, St. Louis, MO, U.S.A.). NPs were prepared by dissolving PLGA (100 mg) in a solution of dichloromethane/trifluoro-ethanol (1:8) containing PFCE (100 mg, Fluorochem, Derbyshire, UK). This solution was then added dropwise to a poly(vinyl alcohol; PVA) solution (5% w/v in water), stirred and sonicated using ultrasonicated probe. Immediately after, the solution was added dropwise in 40 mL Milli-Q water under stirring condition and left to stir for 3 h. NP solution was dialyzed using dialysis membrane (MWCO of 50 kDa) for 3 days against Milli-Q water. NPs were coated with protamine sulfate (PS) in 1:1 (w/w) ratio by agitation at room temperature (RT) for 1 h. After this incubation period, NPs were dialyzed (MWCO of 12 kDa) against Milli-Q water for 3 days, followed by freezing and lyophilization to obtain a dry powder that was stored in a desiccator at RT.

### 2.2 Complexation of NPs and miR

Nanoparticles complexation was performed as described in [21]. Briefly, NPs were weighed and sterilized under ultraviolet light before being resuspended, stirred and sonicated to form a homogenous suspension. For *in vitro* experiments NPs were dissolved to a final concentration of 1 μg/mL in SVZ cell culture medium devoid of growth factors and complexed with a total of 200 nM of miR (50 pmol of miR-124 or scramble-miR, both from GE Healthcare Dharmacon Inc., Chicago, USA) for 45 min at 37 °C with intermittent agitation. For *in vivo* injections, 1 mg of NPs were resuspended into 150 μL of saline solution and complexed with 4 nmol of miR and allowed to complex for 45 min at 37 °C under agitation. Void NPs were prepared using the same procedure but without adding miR. All miR are from GE Healthcare Dharmacon Inc. and were provided annealed, desalted and in the 2’-hydroxyl form and were resuspended in sterile RNA free water.

### 2.3 Zeta potential measurements

PLGA-PS NPs (6.6 mg) were coated with 4 nmol oligonucleotide (similar length as miR-124) for 1 h, at 37°C, and resuspended in 0.9% NaCl solution (1 mL). Zeta potential analyses were performed by light scattering via a Zeta PALS Zeta Potential Analyzer (Brookhaven Instruments Corporation). All data were recorded with at least 5 runs with a relative residual value (measure of data fit quality) of 0.03. To monitor the stability of the NPs (void NPs or NPs conjugated with oligonucleotide; 1.5 mL) under static (no flow) and flow conditions, the NP suspension was stored at 37°C for 24 h or applied to flow conditions in a microfluidic system (Ibidi, μ-Slide VI 0,4 Luer, Germany). In this case, a laminar flow rate of 20 dynes/cm^2^ was applied in the microfluidic system using a perfusion pump system, at 37°C. After 24 h, the NPs were collected, and zeta potential was analyzed.

### 2.4 Primary SVZ cell cultures

C57BL/6 J mice with 1 to 3 day in age were used to obtain SVZ cell cultures as described previously [22]. SVZ fragments were dissected from 450 μm-thick coronal brain sections and placed into HBSS solution supplemented with 100 U/mL penicillin and 100 μg/mL streptomycin (all from Life Technologies, Carlsbad, CA, USA) and digested in 0.025% trypsin, 0.265 mM EDTA (all from Life Technologies), followed by mechanical dissociation. The single cell suspension was diluted in serum-free medium (SFM) composed of Dulbecco's modified Eagle medium [(DMEM)/F12 + GlutaMAX^™^-l)] supplemented with 100 U/mL penicillin, 100 μg/mL streptomycin, 1% B27 supplement, 10 ng/mL epidermal growth factor (EGF), and 5 ng/mL basic fibroblast growth factor-2 (FGF)-2 (all from Life Technologies) and plated onto uncoated petri dishes (Corning Life Science, NY, USA). They were allowed to develop in an incubator with 5% CO_2_ and 95% atmospheric air at 37 °C for five to six days. In these conditions, SVZ cells grow in suspension and generate neurospheres that are rich in neural and progenitor stem cells at distinct stages of differentiation and with proliferative and self-renewing abilities [22–25]. Neurospheres were then seeded onto 0.1 mg/mL poly-D-lysine- (PDL, Sigma-Aldrich Co. LLC) coated glass coverslips in 24-well plates in SFM devoid of growth factors. SVZ cells were allowed to form a cell monolayer for two days before testing the experimental conditions used.

### 2.5 OGD and experimental treatments

**Fig 1:**
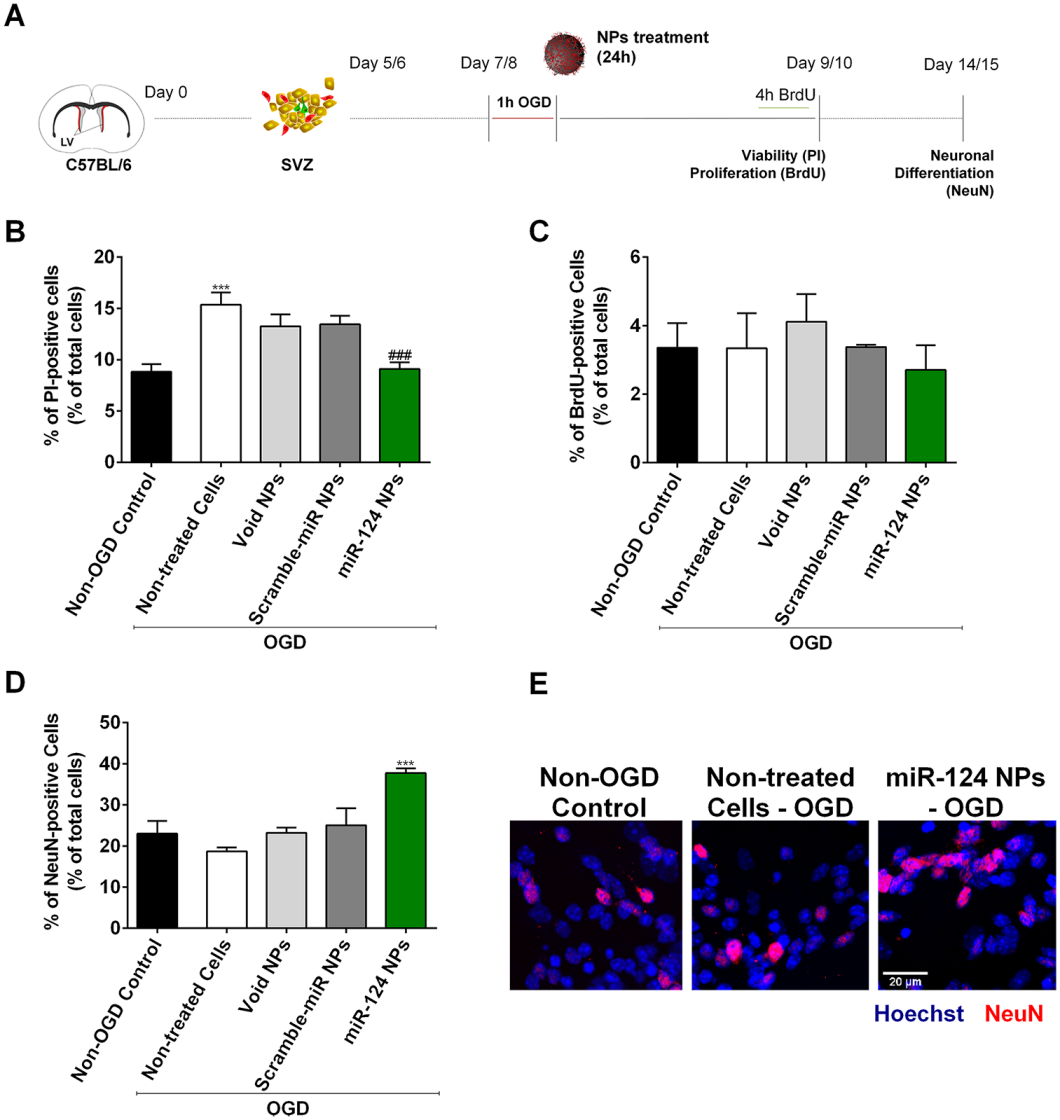
Effect of miR-124 NPs treatment on SVZ cell cultures after OGD. (A) Experimental design of *in vitro* experiments. NSCs where isolated from the SVZ of C57BL/6 J 1 to 3 days-old pups and grown in suspension for 5 or 6 days to obtain neurospheres. Neurospheres were seeded and allowed to grow as monolayer for 2 days before being stimulated with OGD for 1 h. Cells were then incubated with void NPs, scramble-miR NPs or miR-124 NPs for 24 h. Cells were maintained in culture according to the parameters evaluated: 48 h for cell viability and proliferation assays and 7 days for neuronal differentiation. (B) Cell viability assessed by incorporation of propidium iodide (PI) into dead cells and presented as percentage of PI-positive cells in cultures stimulated with OGD and either non-treated or treated with void NPs, miR-scramble NPs or miR-124 NPs, respectively. PI-positive cells quantified in normoxic cultures (non-OGD control) served as controls. (C) Proliferation of SVZ cultures after OGD followed by different treatments. Graphs show the percentage of BrdU-positive cells of total cell counts. D) Neuronal differentiation of the cultures measured by the percentage of NeuN-positive cells in NSC monolayer cultures. (E) Representative fluorescence photomicrographs of NeuN immunostainings in non-OGD control cultures, OGD non-treated and OGD miR-124 NPs treated cultures 7 days after treatment. Nuclei are shown in blue and NeuN in red. Scale bar: 20 μm. Data are expressed as means ± SEM (n = 3). Statistical analysis was performed using one-way ANOVA and Tukey multiple comparison. ***p < 0.001 *versus* non-OGD control; ### p < 0.001 *versus* 1h OGD non-treated cell condition. Abbreviations: NeuN, neuronal nuclei; NPs, nanoparticles; NSCs, neural stem cells; OGD, oxygen and glucose deprivation; SVZ, subventricular zone.

Two days after neurospheres have been seeded the resultant cell monolayer was exposed to OGD for 1 h by replacing the SFM by 0.15 M phosphate-buffered saline (PBS) and incubating the cells in a MIC-101 modular incubator chamber (Billups-Rothenberg Inc., Del Mar, CA, USA) at 37 °C in a 5% CO_2_ and 95% N_2_ gas environment (0.1% O_2_). SVZ cell cultures were then incubated in fresh medium (OGD non-treated cells) or transfected with 1 μg/mL of final concentration of NPs alone or complexed with 200 nM (60 pmol) of miR-124 or scramble-miR (under reoxygenation) in SFM devoid of growth factors for 24 h. A non-OGD control was also used to compare the response of SVZ cells in physiological *versus* OGD conditions. SVZ cells were then allowed to grow as monolayer for two or seven days to analyze cell death and proliferation or neuronal differentiation, respectively (Fig 1A).

### 2.6 Propidium iodide incorporation

SVZ cells were subjected to OGD and then treated with NPs and maintained in culture for 48 h after transfection. Propidium iodide (PI; 5 μg/mL, Sigma-Aldrich Co. LLC) was added for the last 10 min of the 48 h incubation period. Subsequently, cells were fixed with 4% paraformaldehyde (PFA) for 10 min, stained with Hoechst-33342 (4 μg/mL, Life Technologies) for 5 min at RT and mounted in Fluoroshield Mounting Medium (Abcam Plc., Cambridge, UK). Photomicrographs of PI incorporation were taken using an AxioImager microscope (Carl Zeiss, Göttingen, Germany).

### 2.7 5-bromo-2'-deoxyuridine (BrdU) incorporation

SVZ cells were exposed to BrdU (10 μM, Sigma-Aldrich Co. LLC) 4 h before the end of the experiment (48 h post-transfection). Thereafter, cells were fixed in 4% PFA. Fixed cells were permeabilized with 1% Triton X-100 for 30 min at RT and BrdU was exposed by incubating the fixed cells with 1 M HCL for 40 min at 37 °C. Nonspecific binding sites were blocked with 6% bovine serum albumin (BSA) in PBS containing 0.3% Triton X-100 for 1 h, followed by incubation with anti BrdU Alexa-Fluor 594 conjugated antibody (Life Technologies) prepared in PBS containing 0.3% BSA and 0.3% Triton X-100 for 2 h at RT. Cells were then stained with Hoechst-33342 and mounted in Fluoroshield Mounting Medium (Abcam Plc.). Photomicrographs of BrdU incorporation were taken using a confocal microscope (AxioObserver LSM 710, Carl Zeiss).

### 2.8 Neuronal nuclei (NeuN) staining

SVZ cultures were fixed with 4% PFA seven days after being transfected. Cells were permeabilized and blocked for non-specific binding sites in PBS with 0.25% Triton X-100 and 6% BSA for 1 h. Cells were subsequently incubated overnight at 4 °C with a primary mouse monoclonal anti NeuN (1:100, Merck Millipore, Darmstadt, Germany) prepared in PBS with 0.3% BSA and 0.1% Triton X-100. Cells were then incubated for 1 h with the secondary antibody Alexa Fluor 546 donkey anti mouse (1:200, Life Technologies) followed by Hoechst-33342 nuclear staining and mounted in Fluoroshield Mounting Medium (Abcam Plc.). Photomicrographs were taken using an AxioImager microscope (Carl Zeiss).

### 2.9 *In vivo* studies

All experiments were conducted in accordance with protocols approved by the Malmö/Lund Ethical Committee for Animal Research. Nine-week-old C57BL/6 J male mice (Charles River, Sulzfeld, Germany) were housed in the same room and in similar cages under controlled conditions: 12 h light/dark cycle in RT (22 °C) and *ad libitum* access to food and water. Surgeries, animal behavior, histological studies, multiplex immunoassay and *in vivo* statistical analysis were conducted prespecified and in a blinded and randomized manner. All animals were assigned to experimental groups before entering study. A total of 83 mice were used in this study and seven of them died during surgery.

### 2.10 Photothrombotic stroke

Unilateral photothrombotic cortical ischemia in the right primary motor cortex [26] was performed as described previously [27]. Briefly, animals were anesthetized with isoflurane (1.5 to 2% during surgery; Isobeta vet 100%, MSD, AN Boxmeer, Netherlands) and placed into a stereotaxic frame. The local analgesic Marcain (AstraZeneca, Södertälje, Sweden) was injected followed by a scalp incision exposing the mouse skull. The subcutaneous connective tissue was removed and the skull bone was dried. An optic fiber with an aperture of 2 mm per 4 mm was placed in the right hemisphere (center +1.5 mm lateral and +0.5 mm anterior related to Bregma [28] and it was illuminated with a cold light source (Schott KL 1500 LCD, intensity: 3200 K/5D) for 20 min. A photosensitizing dye, Rose Bengal (0.1 mL at 10 mg/mL; Sigma-Aldrich, Taufkirchen, Germany), was injected intraperitoneally 5 min before the illumination. After illumination, the scalp incision was sutured, and the mice were allowed to recover in their home cages. In sham-operated mice, the same procedure was performed but mice were injected with saline solution (0.1 mL of 0.9% NaCl) instead of the photosensitizer. Body temperature was monitored during the surgery and kept between 36 °C and 37.5 °C using a self-regulating heating pad. After surgery, mice temperature and body weight were monitored daily during the experiments and were not altered outside physiological ranges (S1 Table).

### 2.11 Intravenous injections

After surgery, mice were allowed to wake up from anesthesia. Immediately after, mice were placed in a restrainer and injected in the tail vein with a total volume of 150 μL of either saline solution, void NPs, scramble-miR NPs or miR-124 NPs, respectively. Mice were divided into 5 different experimental groups: i) Sham-operated mice (n = 22); ii) PT-operated saline mice (n = 16, 0.9% NaCl); iii) PT-operated void NPs mice (n = 11, 1 mg of NPs); iv) PT-operated scramble-miR NPs mice (n = 12, 1 mg NPs and 4 nmol Scramble-miR); v) PT-operated miR-124 NPs mice (n = 12, 1 mg NPs and 4 nmol miR-124). No differences were found among sham-operated animal treated with saline, void NPs, scramble-miR NPs or miR-124 NPs. To reveal whether the NPs used in the study reached the brain parenchyma, pilot experiments were carried out. After PT, 3 mice were injected either with 1 mg, 5 mg or twice in intervals of 6 h with a dosage of 5 mg of fluorescein isothiocyanate (FITC)-NPs as described above. After 4 or 24 h, mice were perfused with 4% PFA and penetration of NPs was evaluated in coronal sections by fluorescence microscopy.

### 2.12 Behavior analysis

For behavior analysis, animals subjected PT surgery (considered for infarct volume assessment; section 2.13) or sham surgery where considered excepted for mice with a score below 3 on day -1 (the day before surgery), which were not included in assessment of recovery of function after surgery (1 mice excluded in a total of 46).

#### 2.12.1 Rotating pole test

Rotating pole test was performed as described previously [29]. Mice were trained for 2 days to traverse an elevated wooden pole (750 mm above ground, diameter 15 mm, length 1,500 mm) that was rotating at 0, 3 or 10 rotations *per* min (rpm) to the right or left. Mice were evaluated on the day before surgery (day -1) and on days 2, 7 and 14 after surgery. Mice were attributed a score from 0 to 6: 0, the mouse falls off the pole immediately upon placement of the pole; 1, the mouse is unable to traverse the pole remaining sitting or trying to go backwards; 2, the mouse falls off the pole while crossing or the hind limbs do not contribute to forward movement; 3, the mouse manages to reach the platform but it is constantly slipping with the fore- and/or hind limbs; 4, the mouse traverses the pole with more than four slips; 5, the mouse crosses the pole with one to three slips; 6, the mouse traverses the pole with no foot slips.

#### 2.12.2 Grid test

Mice were trained to cross a grid (600 mm length) before surgery. Each mouse was evaluated before surgery (day 0) and at days 2, 7 and 14 after surgery. Each misstep of the paretic paws was counted as one fault and the number of faults was scored from 0 to 6: 0–6 and more faults; 1–5 faults; 2–4 faults; 3–3 faults; 4–2 faults; 5–1 fault; 6 –no fault.

### 2.13 Tissue collection

Mice were euthanized at two time points: 2 days after PT for protein extraction and serum collection (analysis of pro-inflammatory cytokines, total of 27 mice) and 14 days after stroke for immunohistochemistry (infarct volume and neurogenesis, total of 46 mice). At 2 days after surgery, animals were deeply anesthetized with isoflurane and subjected to a thoracotomy to expose the heart to collect a blood sample from the left ventricle. Samples were centrifuged at 10,600 g for 5 min and serum was stored at -80 °C until further use. After blood collection, mice were decapitated and the brain was dissected and immediately frozen in isopentane (Sigma-Aldrich, Taufkirchen, Germany) on dry ice. The ischemic territory (infarct core and proximal peri-infarct tissue) was then dissected from the mice brains in a glove box at -20 °C to avoid protein degradation. Thereafter, brain tissue was stored at -80 °C until further processing. At 14 days after surgery mice were deeply anesthetized with pentobarbital and perfused intracardially with saline solution followed by ice cold 4% PFA (pH 7.4, Sigma-Aldrich). Brains were removed and post-fixed with 4% PFA for 24 h, followed by immersion in a 25% sucrose solution (Sigma-Aldrich). Thereafter, brains were sectioned on a microtome (SM 2000R, Leica Microsystems, Wetzlar, Germany) at 30 μm-thick coronal sections. For infarct volume measurement sections spaced 460 μm to each other were collected from the start until the end of the lesion. Sections were stored at -20 °C in an antifreeze solution made in PBS containing 30% glycerol and 30% ethylene glycol. To analyze the penetration of NPs into the brain parenchyma mice were euthanized 4 or 24 h after injection of FITC-NPs and fixed in 4% PFA as described above.

### 2.14 Infarct volume

For each PT operated animal, the 30 μm-thick coronal sections, distanced 460 μm from each other were stained against the neuronal marker NeuN. Stained sections were digitalized at 9600 dpi (CanoScan 8800F, Canon, Tokyo, Japan) and processed with the ImageJ software (National Institute of Health, USA). For each animal, the infarct volume (mm^3^) was calculated by subtracting the area of the non-lesioned ipsilateral hemisphere from the area of the intact contralateral hemisphere followed by their volumetric integration. Mice whose infarct volume was lower than 0.2 mm^3^ or higher than 6.0 mm^3^ were excluded from the study (7 mice excluded in a total of 46).

### 2.15 Immunohistochemistry/Immunofluorescence

Free-floating brain sections were rinsed three times in PBS followed by the quenching of the endogenous peroxidases activity with 3% H_2_O_2_ (Sigma-Aldrich) for 20 min at RT. Slices were then rinsed three times for 10 min each with PBS and sections were permeabilized and blocked using 5% normal donkey serum (Jackson ImmunoResearch Laboratories Inc., Suffolk, UK) dissolved in PBS-T (PBS with 0.25% of Triton X-100) for 1h at RT. Sections were incubated overnight at 4 °C with a rabbit anti NeuN (Millipore, 1:5000) in PBS-T with 3% normal donkey serum. Thereafter, sections were rinsed in PBS-T three times and incubated in biotinylated secondary antibody donkey anti rabbit (Jackson ImmunoResearch Laboratories Inc.) at a dilution of 1:400 in PBS-T with 2% normal donkey serum for 90 min at RT. Visualization was achieved *via* the Vectorstain ABC kit (Vector) using 3,3-diaminobenzidine-tetrahydrochloride (DabSafe, Saveen Werner, Sweden), 8% NiCl_2_ and 3% H_2_O_2_. Sections were dehydrated in consecutive higher concentrations of ethanol, 2 min in 70% ethanol, two times 2 min in 95% ethanol, 2 times 2 min in absolute ethanol, followed by two times 2 min in xylol and mounted using Pertex (Histolab AB, Gothenburg, Sweden). For fluorescence stainings, free-floating sections were rinsed three times in PBS and incubated with 2 M HCl for 25 min at 37 °C to induce DNA denaturation and exposure of BrdU. Sections were then incubated in blocking solution– 2% of normal donkey serum and 0.3% Triton X-100 in PBS—for 2 h at RT, followed by a 48h incubation overnight at 4°C using the following primary antibodies: rat monoclonal anti BrdU (1:250, AbD Serotec, Raleigh, NC, U.S.A.) and goat polyclonal anti doublecortin (DCX; 1:1000, Santa Cruz Biotechnology, Inc., Santa Cruz, U.S.A.) Thereafter, sections were incubated with anti rat biotin-SP secondary antibody (1:200, Jackson ImmunoResearch Laboratories Inc.) and DAPI (1:10,000) for 1h at RT followed by incubation with donkey streptavidin Alexa Fluor 488 and Cy3 donkey anti goat (all 1:200, from Jackson ImmunoResearch Laboratories Inc.) for 2 h. Then, a simplified version of this protocol was used to study penetration of NPs into brain parenchyma. Briefly, 30 μm thick coronal sections were incubated in blocking solution for 1 h at RT and then incubated with the goat polyclonal anti FITC (1:500, Abcam Plc.) in combination with mouse monoclonal anti CD31 (1:1000, Abcam Plc.) or rabbit monoclonal anti NeuN (1:1000, Cell Signaling Technology, Leiden, Netherlands) or rabbit polyclonal anti GFAP (1:20000, Dako, Glostrup, Denmark) for 48 h at 4 °C. Thereafter, sections were incubated with Hoechst-33342 (1:10,000) and the secondary antibodies, Alexa Fluor 488 conjugated donkey anti goat and Alexa Fluor 546 conjugated donkey anti mouse (both 1:1000, Life Technologies) for 2h at RT. Finally, sections were mounted in Fluoroshield Mounting Medium (Abcam Plc.). Photomicrographs were obtained using an LSM 510 or AxioObserver LSM 710 confocal microscope (Carl Zeiss).

### 2.16 Preparation of protein extracts

Whole protein extracts were obtained from the ischemic territory and corresponding regions in sham animals. Tissue homogenization was done by sonication (Cole Parmer Instruments Co., Chicago, U.S.A.) in lysis buffer containing 20 mM Tris (pH 7.5), 150 mM NaCl, 1 mM EDTA, 1 mM ethylene glycol tetraacetic acid (EGTA), 1 mM phenylmethanesulfonyl fluoride (PMSF), 2.5 mM sodium pyrophosphate, 1 mM β-glycerolphosphate, 1 mM sodium orthovanadate supplemented with protease inhibitor cocktail. Thereafter, tissue was incubated on ice for 10 min followed by a centrifugation (20,000 g at 4 °C for 20 min) and the supernatant was collected for further analysis. Concentration of the whole protein collected was done by the Bradford assay using BSA (Sigma-Aldrich) dissolved in lysis buffer as standard.

### 2.17 Cytokine analysis from brain extracts and serum samples

The levels of different cytokines—namely interferon-gamma (IFNγ), interleukin-1beta (IL-1β), IL-6 and tumor necrosis factor-alpha (TNF-α)–were evaluated using a multiplex immunoassay kit according to the manufacturer’s protocol (Mesoscale, Gaithersburg, MD, USA) in the five experimental groups tested. For this evaluation 50 μg of total protein from the protein extracts and 50 μL of serum collected from the same mice were used.

### 2.18 Statistical analysis

Analyses of immunocytochemistry experiments were performed at the border of seeded neurospheres where cells formed a pseudo-monolayer. The experiments were performed in six independent cultures obtained from three different SVZ cell isolations from C57BL/6 pups. Percentage of PI-positive, BrdU-positive and NeuN-positive cells were calculated from cell counts in five independent microscopic fields (approximately 150 cells *per* field) from each coverslip with a 40x magnification. *In vivo*, quantification of DCX-positive, BrdU-positive and DCX/BrdU-double positive cell number was performed in the SVZ and in the peri-infarct cortex of at least 2 animals, in 30 μm coronal sections located +1 mm to bregma. Cells were counted along the slice thickness obtained by Z-stacks (40x magnification). To obtain an unbiased density estimate, fields with the same mean total volume and similar total number of SVZ cells were selected.

The software used for cell counting was ImageJ (NIH Image, Bethesda, MD, USA). Data are expressed as mean ± standard error of mean (SEM) or as medians with the 1^st^ and 3^rd^ quartile and whiskers. Statistical analyses have been performed with GraphPad Prism 6 software (GraphPad, San Diego, CA, USA) by using ANOVA followed by Tukey multiple comparison test (parametric values) or by Kruskal Wallis analysis followed by Dunn´s post hoc evaluation (non-parametric values), with p<0.05 considered to represent statistical significance.

## 3. Results

### 3.1 miR-124 NPs protect SVZ cells and stimulate their differentiation after OGD

SVZ cells have been isolated and grown *in vitro* as neurospheres (cell suspensions) that are mostly stem/progenitors cells. A polymeric NP formulation was used as a carrier to deliver miR-124 into SVZ cells. These NPs have a MRI tracer (perfluoro-1,5-crown ether) in their core and are mainly constituted of a polymer—PLGA—that is coated with a cationic agent, protamine sulfate, to allow the complexation of negatively charged miR molecules. Based on our previous studies demonstrating that 1 μg/mL of NPs complexed with 200 nM (60 pmol) of miR-124 induced neuronal differentiation of SVZ cells *in vitro* without cytotoxic effects, we applied these conditions to evaluate if miR-124 NPs have a beneficial effect on SVZ cells exposed to a combined oxygen and glucose deprivation. SVZ cultures obtained from post-natal C57BL/6 J mice were exposed to OGD for 1 h followed by a reoxygenation period of 24 h with either fresh medium (non-treated cells) or fresh medium containing miR-124 NPs or scramble-miR NPs or void NPs. Control cells were not subjected to OGD (normoxic non-OGD control). Exposure to OGD for 1 h led to a significant 1.7-fold increase in the cell death of SVZ cultures, being this effect reverted in cultures transfected with miR-124 NPs after the OGD insult (Fig 1B; non-control OGD 8.82 ± 0.76, non-treated cells 1 h OGD 15.36 ± 1.19, miR-124 NPs 1 h OGD 9.10 ± 0.65, ***p<0.001, ###p<0.001; data are presented as percentages of total number of cells). To measure cell proliferation BrdU was incubated with the SVZ cells for the last 4 h of the 48 h post-OGD incubation period. BrdU is a thymidine analog that incorporates into the DNA of cells during the S phase of the mitotic process. The total levels of BrdU-positive cells were not altered by the exposure to OGD *per se* nor by the presence of miR-124 NPs after OGD (Fig 1C). Finally, we studied the miR-124 NPs neurogenic potential after OGD by staining the cultures with the mature neuronal marker NeuN (Fig 1D and 1E). Surprisingly, OGD exposure did not alter the levels of neurons obtained from SVZ cultures when compared with non-OGD basal conditions. Nevertheless, the presence of miR-124 NPs after the OGD insult resulted in a 1.6-fold increase in the NeuN-positive cells compared with non-OGD control (Fig 1D and 1E; non-OGD control 23.03 ± 3.08, non-treated cells OGD 18.69 ± 0.99; miR-124 NPs 1h OGD 37.74 ± 1.12, ***p<0.001; data are presented as percentages of total number of cells). These results indicate that miR-124 NPs may not only have a neurogenic potential but are also neuroprotective after OGD *in vitro*.

### 3.2 Treatment with miR-124 NPs does not affect lesion volume and functional outcome after photothrombosis

**Fig 2:**
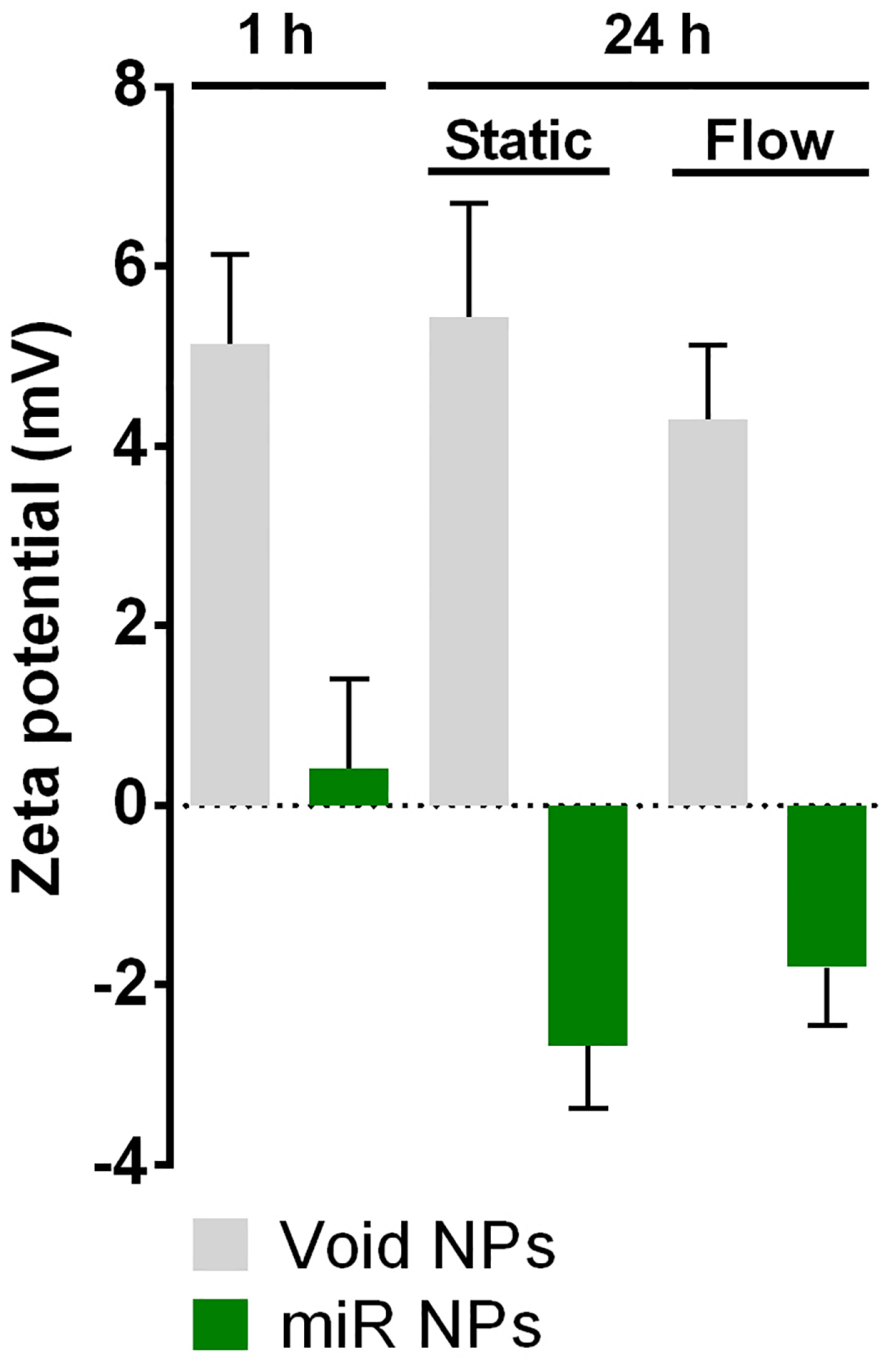
NP formulation are stable for 24 h. Bar graph shows zeta potential (mV) from void NPs or oligonucleotide-loaded NPs (miR NPs) at 1 h and 24 h after complexation, both in static and flow (20 dyn/cm^2^) conditions. Abbreviation: NPs, nanoparticles.

Next, we aimed at evaluating if administration of miR-124 NPs could modulate processes in the post-ischemic brain contributing to recovery of lost neurological function. First, we analyzed stability of the NP formulation for 24 h both in static conditions and at flow conditions, in order to mimic the flow found in blood vessels (Fig 2). Zeta potential measurements showed no changes in zeta potentials of void NPs after 24 h either in static or flow conditions, demonstrating the stability of the formulation. In case of miR NPs formulation, a decrease in the zeta potential after 24 h (Zeta potential: -2.7±1.2 mV (flow); -1.8±1.1 mV (static)) relatively to the initial formulation (Zeta potential: 0.4±1.7 mV) was observed (Fig 2). This process occurred both in static and flow conditions, likely due to the adsorption of salts or oligonucleotide that remained in solution when the stability tests were initiated.

**Fig 3:**
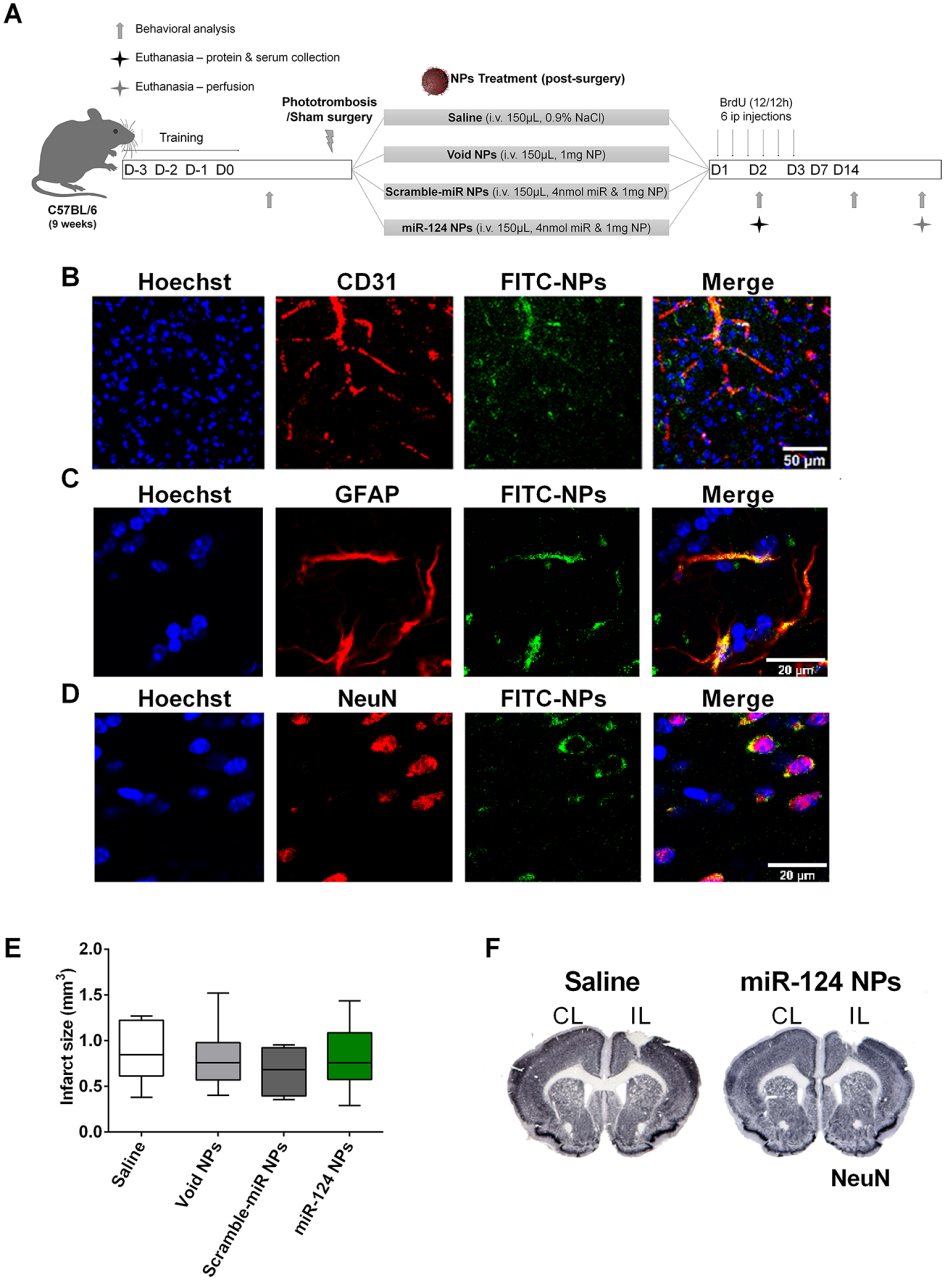
miR-124 NPs do not affect infarct volume of PT mice. (A) Experimental design for the *in vivo* experiments. Before entering the study, mice were assigned to surgery and treatment groups subjecting them either to sham surgery or PT. The PT group was then subdivided into 4 subgroups in which mice were treated with an intravenous injection in the tail vein of saline, void NPs, scramble-miR NPs or miR-124 NPs (total of 5 different groups) immediately after surgery. Pro-inflammatory cytokines have been measured on day 2 after surgery from the ischemic core and adjacent peri-infarct tissue and serum. Infarct volumes and neurogenesis have been studied 14 days after surgery. These mice received BrdU injections (every 12 h) during the first 3 days after surgery. After a training period, neurological function was tested on days -1, 2, 7 and 14, respectively. (B, C, D) Representative photomicrographs of the peri-infarct area 4 h after intravenous injection of 1 mg of FITC-NPs (green) and stained against the nuclear marker Hoechst-33342 (blue) and either the endothelial marker CD31 (red) (B), or the astrocyte marker GFAP (red) (C), or the neuronal marker NeuN (red) (D). Scale bar 50 μm. (E) Infarct volume (in mm^3^) in the 4 different groups following PT. Data are expressed as medians with the 1^st^ and 3^rd^ quartile with the following number of animals included in each experimental group: PT saline n = 6, PT void NPs n = 6, PT scramble-miR NPs n = 8, PT miR-124 NPs n = 8. F) Representative images of the infarct area (white) in coronal sections stained with NeuN of mice treated with saline (left) or miR-124 NPs (right). Abbreviations: BrdU, 5-bromo-2'-deoxyuridine; CL, contralateral hemisphere; GFAP, glial fibrillary acidic protein; IL, ischemic hemisphere; NeuN, neuronal nuclei; NPs, nanoparticles; PT, photothrombosis.

Then, we evaluated sensorimotor function, the inflammatory response (pro-inflammatory cytokine levels in the ischemic territory and periphery) and neurogenesis (DCX/BrdU cell number in SVZ and peri-infarcted area) in mice subjected to PT or sham operation. Behavioral tests were performed on day -1 or day 0 and day 2, day 7 and day 14 after surgery. Following PT or sham surgery, the inflammatory response was studied on day 2 post-stroke while neurogenesis was evaluated on day 14 in mice that were intravenously injected with miR-124 NPs, scramble-miR NPs, void NPs or saline, respectively (Fig 3A). All mice were monitored daily in terms of body weight and temperature, no differences were observed between the treatment groups (S1 Table).

In pilot experiments, we observed that NPs were able to penetrate into the brain parenchyma by injecting 1 mg of FITC-NPs to mice immediately after PT. Co-staining of the FITC-NPs with the marker for endothelial cells CD31 clearly showed a wide distribution of FITC immunoreactivity throughout the brain parenchyma 4 h after intravenous injection. Signals were observed in brain microvessels as expected, indicating that there was not a complete penetration of NPs (Fig 3B). Importantly, FITC-NPs were found in the striatum and cortex, including the peri-infarcted area; in addition, significant accumulation of FITC-NPs was observed in white mater, namely the corpus callosum and taken up by astrocytes (Fig 3C) and neurons (Fig 3D). Administration of higher dosages (5 and 10 mg) of FITC-NPs showed similar results at 4 and 24 h following intravenous injection.

Evaluation of infarct volumes revealed no differences between all treatment groups: PT saline 0.87 ± 0.14, PT void NPs 0.81 ± 0.15, PT scramble-miR NPs 0.67 ± 0.09, PT miR-124 NPs 0.81 ± 0.13 (Fig 3E). As shown in Fig 3F representative coronal sections show similar infarct areas in mice treated either with saline or miR-124 NPs, indicating that miR-124 NPs do not contribute to a reduction of the ischemic lesion. From these results, we can exclude that differences in lesion volumes did not influence outcome measures.

**Fig 4:**
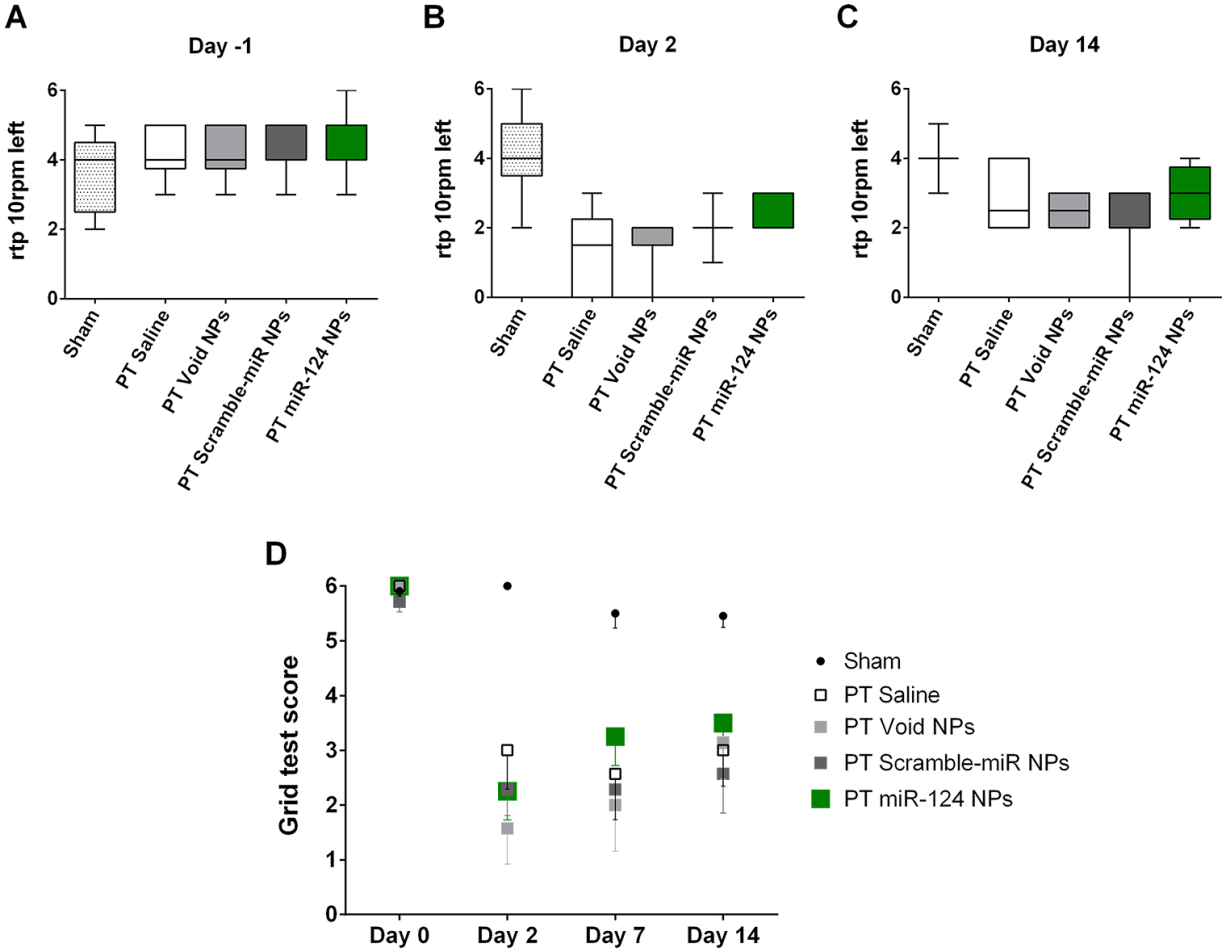
miR-124 does not affect neurological function after photothrombosis. (A-C) Rotating pole test scores of mice subjected to sham surgery or PT surgery and treated either with saline, void NPs, scramble-miR NPs or miR-124 NPs administered intravenously immediately after PT. Results in (A-C) show the performance at 10 rotations of the pole to the left a day before surgery (day -1), 2 days and 14 days after the insult, respectively; data are presented as medians with the 1^st^ and 3^rd^ quartile. (D) Grid test presenting the number of foot faults of the left side paws traversing a 60 cm long grid as indicated in the Methods section. Sham-operated mice and mice subjected to PT of the abovementioned treatment groups were evaluated at days 0, 2, 7 and 14. All data are represented as medians with the 1^st^ and 3^rd^ quartile with 6 to 11 animals per group: sham n = 11, PT saline n = 6, PT void NPs n = 6, PT scramble-miR NPs n = 7, PT miR-124 NPs n = 8. Abbreviations: NPs, nanoparticles; PT, photothrombosis.

Neurological deficits and their recovery were assessed by two independent behavioral tests: the rotating pole test (Fig 4A–4C) and the grid test (Fig 4D). After PT, mice showed neurological deficits that were not evident before PT. Prior to PT, all animals performed the rotating pole test with a median of 4 points (Fig 4A). On day 2, the majority of the animals could not traverse the pole, the median throughout the groups was 2 (Fig 4B). No difference was observed comparing the four treatment groups. A slight but non-significant recovery has been observed 14 days after PT due to spontaneous recovery. Similar to day 2 treatment with miR-124 NPs did not improve motor function in mice subjected to PT (Fig 4C) compared to the non-treated (saline) mice subjected to PT. Likewise, mice subjected to PT made a significant higher number of foot faults in the grid test. Also here, treatment did not affect the performance at any time point of measurement (Fig 4D). In both behavioral tests sham-operated mice did not show deficits. They had a similar performance throughout the study (Fig 4A–4D).

### 3.3 SVZ Neurogenesis after miR-124 NPs treatment in PT mice

**Fig 5:**
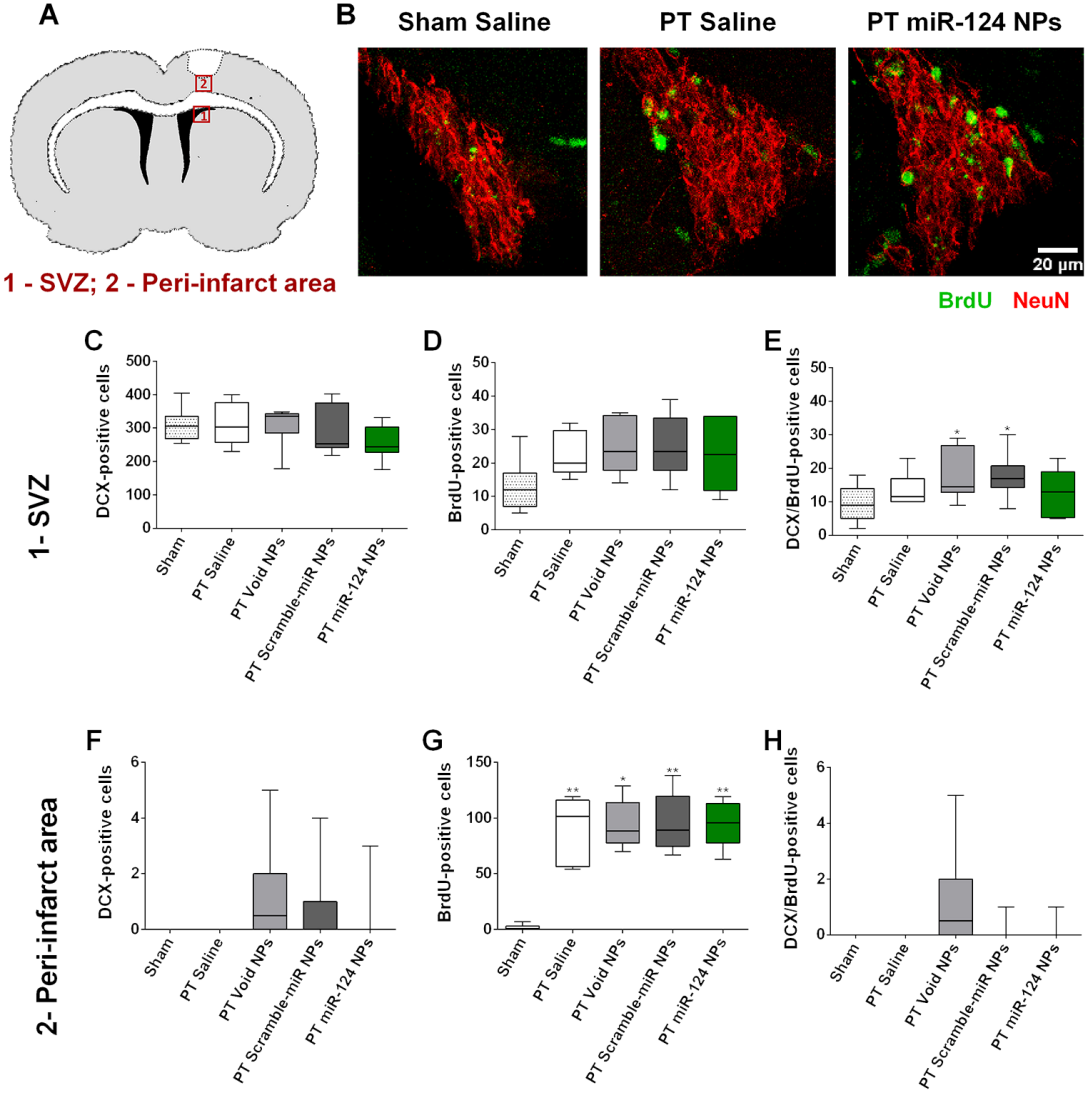
Neurogenesis is not affected by miR-124 NPs treatment. (A) Illustration of a coronal slice of the mouse brain representing the areas used to evaluate neurogenesis in the SVZ (red rectangle 1) and the peri-infarct area (red rectangle 2). (B) Representative confocal images of BrdU (green) and DCX (red) staining observed in the SVZ of sham-operated animal, a mouse subjected to PT and saline-treated and a mouse subjected to PT and miR-124 NPs-treated, respectively. Scale bar: 20 μm. Total number of (C, F) doublecortin (DCX)-positive cells, (D, G) BrdU-positive cells and (E, H) DCX/BrdU-double positive cells in the SVZ (C-E) and peri-infarct area (F-H) of mice after sham surgery or PT and indicated treatment conditions. All data are expressed as medians with the 1^st^ and 3^rd^ quartile values, with the following number of animals included in each experimental group: sham n = 11, PT saline n = 6, PT void NPs n = 6, PT scramble-miR NPs n = 8, PT miR-124 NPs n = 8. *p < 0.05 *versus* sham group and **p < 0.01 *versus* sham group. Abbreviations: BrdU, 5-bromo-2'-deoxyuridine; NPs, nanoparticles; PT, photothrombosis; SVZ, subventricular zone.

To evaluate neurogenesis, mice were injected intraperitoneally with BrdU for 3 days after surgery (every 12 h, Fig 3A) in order to assess dividing cells. The number of cells positive for DCX (marker of neuroblasts), BrdU and double positive for DCX and BrdU were evaluated in the SVZ (Fig 5A–5E) and peri-infarct area (Fig 5A and 5F–5H). In the SVZ, two weeks after surgery and miR-124 NPs injection, we did not observe any differences between the number of DCX-positive cells in the PT animals when compared with sham-operated mice nor among the different treatment groups in PT animals (Fig 5C). We observed that PT animals tend to have slightly higher numbers of BrdU-positive cells compared to sham-operated animals (Sham 12.55 ± 1.98; PT saline 22.33 ± 2.73; PT void NPs 24.83 ± 3.38; PT scramble-miR NPs 25.13 ± 3.28; PT miR-124 NPs 22.63 ± 3.80; medians with the 1st and 3rd quartile). Hence, treatment with miR-124 NPs did not change the total number of BrdU stained cells when compared with saline, void NPs or scramble-miR NPs, respectively (Fig 5D). This increase, however, can be explained by higher levels of non-neuronal cells that may proliferate, as well as cell death in response to the damage caused by the PT. Likewise the number of DCX-positive cells and DCX/BrdU-double positive cells were also not altered among the five experimental groups (Fig 5C and 5E). To investigate the number of cells that potentially migrated from the SVZ to the lesion area, a region of interest was created located above the SVZ and underneath the infarcted area (Fig 5A), or the equivalent region in sham animals, was evaluated. Sham mice did not present any DCX-positive cells and the number of BrdU-positive cells was negligible (Sham 1.82 ± 0.69; medians with the 1st and 3rd quartile). Regarding the PT mice, the number of BrdU positive cells was elevated in all the groups independent of the treatment (Fig 5G; PT saline 91.33 ± 11.90; PT void NPs 94.17 ± 8.77; PT scramble-miR NPs 95.25 ± 8.90; PT miR-124 NPs 94.75 ± 6.88; medians with the 1st and 3rd quartile). Despite some reports suggesting the migration of SVZ neuroblasts into the peri-infarcted area [30,31] we found very few DCX-positive cells in this area (Fig 5H; PT saline 0.00 ± 0.00; PT void NPs 1.17 ± 0.79; PT scramble-mIR NPs 0.75 ± 0.49; PT miR-124 NPs 0.38 ± 0.38; medians with the 1st and 3rd quartile).

From these results, we conclude that stroke induced neurogenesis does not exist in our experimental conditions and that a single intravenous injection of miR-124 NPs was unable to elicit an increase in SVZ neurogenesis neither in the healthy nor in post-stroke brain.

### 3.4 Effects of miR-124 on the post-ischemic inflammatory response

**Fig 6:**
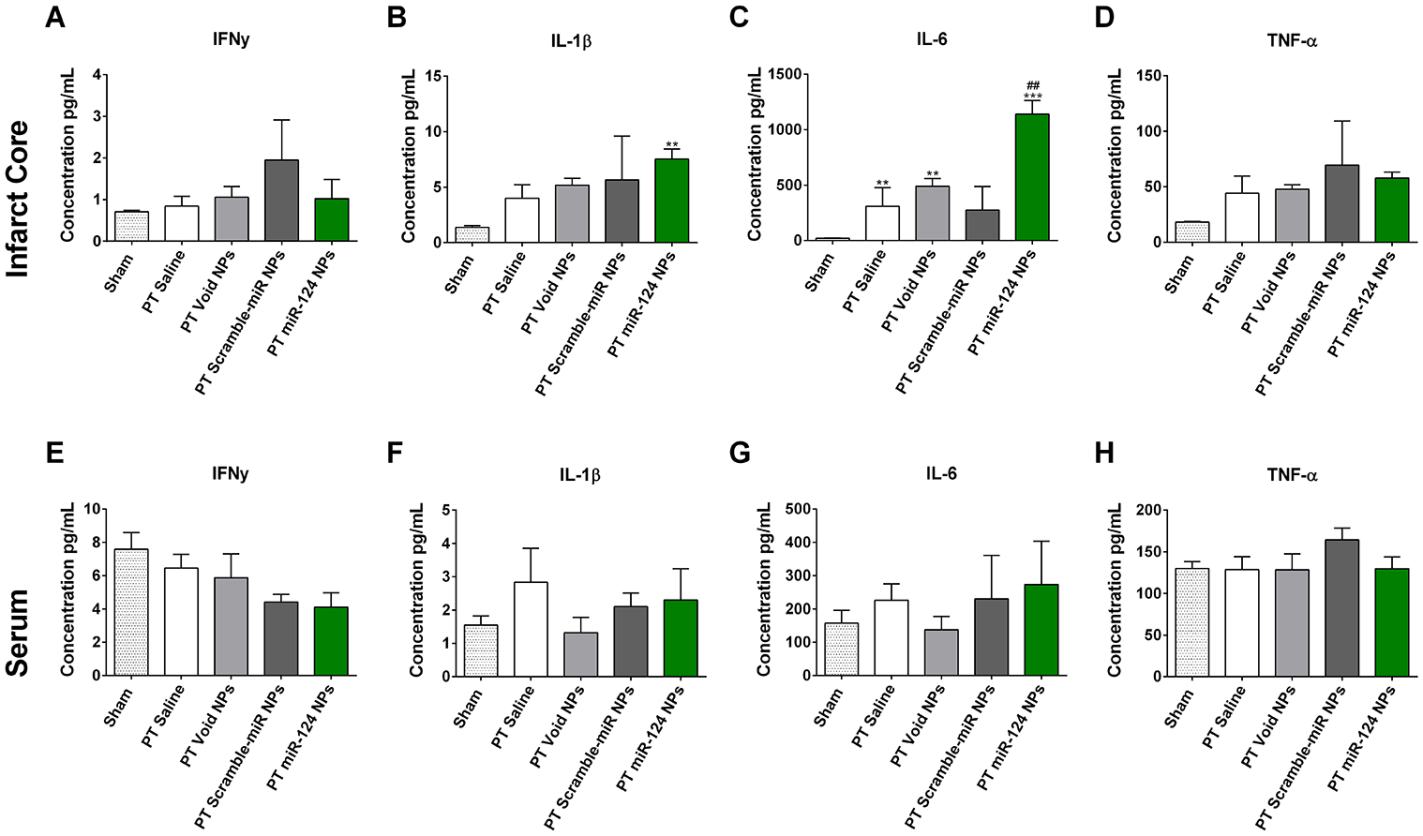
Effects of miR-124 NPs on levels of pro-inflammatory cytokines in the ischemic territory and serum. (A-D) Levels of IFN-γ, IL-1β, IL-6 and TNF-α in the ischemic territory (infarct core and adjacent peri-infarct tissue) 2 days following PT. (E-H) Serum levels of the above mentioned cytokines from the same animals. Data are shown as mean ± SEM with the following number of animals included in each experimental group: sham n = 10, PT saline n = 4, PT void NPs n = 4, PT scramble-miR NPs n = 3, PT miR-124 NPs n = 4. ** indicates p < 0.01 and *** indicates p < 0.001 *versus* sham group, ^##^ indicates p < 0.01 *versus* all other experimental conditions after PT. Abbreviations: NPs, nanoparticles; PT, photothrombosis.

miR-124 is predicted to attenuate inflammatory pathways, since a reduction in the miR-124 is needed to obtain a reactive microglial state [32]. We measured the levels of pro-inflammatory cytokines, namely IFNγ, IL-1β, IL-6 and TNF-α 48 h after PT (Fig 6). The levels of these four cytokines were measured in the ischemic territory (infarct core and peri-infarct area) to evaluate the local inflammatory response as well as in the serum of the same mice. In the brain, we found an elevation of all four cytokines after PT compared to sham-operated mice, however, only IL-6 reached statistically significant levels. Animals subjected to PT treated with miR-124 NPs showed significantly higher levels of IL-6 compared to all experimental groups (Fig 6C). Regarding the peripheral response, there was no difference between any of the five experimental groups (Fig 6E–6H), supporting previous studies that a peripheral immune response may be transient and limited to the first hours after stroke onset [33,34]. Together, miR-124 NPs were able to increase IL-6 levels although no changes in the other pro-inflammatory cytokines evaluated were observed.

## 4. Discussion

In this study, we investigated the effect of intravenous administration of miR-124 NPs as a possible therapeutic strategy to improve stroke outcome following permanent focal ischemia induced by photothrombosis.

Endogenous NSCs participate in neural regeneration after injuries, namely in cerebral ischemia [35]. miR-124 is the most abundant microRNA in the adult brain [36] and it is a well described inductor of SVZ neurogenesis [37,38]. Indeed, we have recently shown that the delivery of miR-124 into the lateral ventricles potentiated SVZ neurogenic potential leading to the amelioration of motor symptoms in mice lesioned with 6-hydroxydopamine [21]. Herein, we first showed that SVZ cells, which were exposed to OGD, and then treated with miR-124 NPs were protected and prone to differentiate towards a neuronal phenotype. These results prompted us to test the efficacy of miR-124 NPs *in vivo* in a permanent focal photothrombosis mouse model for ischemia. In this study, the miR-124 NPs were administered systemically since the intracerebroventricular injection of drugs is not a viable option for the majority of stroke patients. However, one of the major problems is the penetration into brain parenchyma through the blood brain barrier (BBB) [39]. Nevertheless, after a PT stroke the BBB is affected and becomes more permissive. In rodents subjected to PT stroke, extravasation of the Evans blue dye was seen in the brain parenchyma as soon as 2 h after the ischemic event reaching the maximum at 12 h and 24 h followed by a gradual decrease [40,41]. Moreover, higher matrix metallopeptidase- 9 (MMP-9) levels and activity as well as increased putative transcytosis of vesicles and vacuole were seen in the endothelium of rodents after PT stroke [40–42]. These alterations may aid the miR-124 NPs passage through the BBB. Another important aspect to consider in miR-based therapeutics is dosage. High dosages of miRNA can result in saturation of the miRNA machinery leading to cellular toxicity, off-target effects that can cause toxicity in non-target tissues, and immunological response that can be harmful for the recipient [43]. NPs are attractive vehicles to transport molecules into the CNS due to: high load of cargo, ability to protect the cargo, high stability both *in vivo* and storage, and easiness to be modulated in terms of size, shape, surface and chemistry. Our formulation showed to be stable throughout 24 h, with void NPs showing no significant alterations when maintained at 37 °C both in static and flow conditions, indicating that they can be useful carriers for systemic delivery. In fact, herein we show the internalization of NPs into brain resident cells. These proof-of-principle experiments unequivocally demonstrate the delivery of miR-124 loaded NPs into the post-ischemic brain. However, miR-124 NPs did not improve functional outcome after the insult. We observed similar infarct volumes between experimental groups to exclude infarct volume as a possible confounding variable for outcome measures. Regarding neurogenesis, we observed that the total number of DCX/BrdU-double positive cells was also not altered in the SVZ. In addition, we found an increased number of BrdU-positive cells in the ischemic territory. However, almost no DCX/BrdU-double positive cells were detected in the ischemic core and peri-infarct tissue. These results may indicate that lack of efficiency might be due to dosage, timing and route of administration of miR-124 important for its bioavailability and need. In fact, as mentioned before we have observed that a intracerebroventricular injection of miR-124 NPs results in enhancement of olfactory bulb neuroblasts under physiological conditions in mice four weeks after injection [21]. Moreover, we showed that 24 h after transfection both miR and NPs could be found throughout the cytoplasm of cells, while only 5% were located in late endosome and lysosome vesicles [20,21], indicating that the formulation we used allows miR release inside the cells and avoids endolysosomal degradation. miR was also efficiently localized with the Argonaute 2 protein, an essential component of the microRNA-mediated silencing complex (miRISC) [20].

Previously, it has been shown that viral administration of miR-124 for 21 days prior to MCAO resulted in a decrease of infarct volumes, reduced microglial activation and enhanced motor behavior [14]. Moreover, local injection of miR-124 in the striatum of mice subjected to transient MCAO two days after stroke resulted in a reduction of lesions and better functional outcome [17]. On the other hand, infusion of an inhibitor of miR-124 (antagomir) into the lateral ventricle of rats prior to transient MCAO resulted in smaller infarct volumes with a significant reduction of TUNEL-positive cells [18]. Another study even reports that intraventricular administration of miR-124 in mice immediately after MCAO resulted in a non-significant increase of infarct volumes 24 h after stroke [19]. It seems that the route of administration of miR-124 is critical to achieve biological effects of miR-124 in the post-ischemic brain. Also, it needs to be considered that at an early time point following an ischemic insult the infarct has not been subsided. Therefore, effects of miR-124 on lesion volumes at an early stage following stroke i.e. 24 h might be due to effects on multiple processes i.e. brain edema without consequences for the final ischemic lesion and function [44]. Also, differences in results might be due to different experimental stroke models. With some reservations, it seems that miR-124 is rather efficient in transient stroke models than in permanent models. Also, further studies need to elucidate the exact role of miR-124 antagonism in the post-ischemic brain. Our results do not support our initial hypothesis that miR-124 NPs could improve functional outcome following PT, with no changes in behavioral tests observed in this study.

The inflammatory response is a consequence of stroke and it contains well-orchestrated cascades involving resident and peripheral immune cells together with the expression and release of inflammatory molecules [45]. miR-124 reduction is needed in order to shift the resting state of microglia to a reactive state [32]. miR-124 seems to be essential for the activation and maintenance of the M2 inflammatory state of microglia, through the modulation of the C/EBP-α-PU.1 pathway [46–49], the USP2 and USP14 [50], the signal transducer and activator of transcription 3 (STAT3), TNF-α converting enzyme (TACE), necrosis factor (NF)-κB p65, TRAF6 [51,52], among other inflammatory mediators. However, studies have shown that miR-124 does not always act as a repressor for microglia activation [53,54]. For example, in epilepsy Brennan et al. have proved that status epilepticus leads to suppressed miR-124 expression, while administration of synthetic miR-124 significantly augments microglia activation and inflammatory cytokines, including IL-1β, TNF-α and IL-6. In fact, they observed a similar effect (higher microglia reactivity) also in control mice treated with miR-124 [53]. In Crohn’s disease miR-124 promotes intestinal inflammation. The authors verified that miR-124 overexpression resulted in the production of pro-inflammatory cytokines by targeting the aryl hydrocarbon receptor [54]. In fact, aryl hydrocarbon receptor is able to suppress IL-6 production through inhibition of histamine production in LPS-stimulated macrophages [55]. Importantly, the only pro-inflammatory cytokine modulated by miR-124 NPs after PT was IL-6. Elevated levels were exclusively found in miR-124 NPs animals following PT suggesting a specific miR-124 mediated effect. This is in contrast to what has been observed before and may rather support a pro-inflammatory role of miR-124 as reported in models of epilepsy [53]. On the other hand, administration of miR-124 NPs to mice subjected to PT did not affect the levels of IL-1β, IFNγ and TNF-α. Also, no significant peripheral inflammatory response was seen among sham- and PT-operated animals nor among the different treatments. This goes along with previous studies questioning a prolonged upregulation of pro-inflammatory cytokines in rodents following stroke [33,34].

For the systemic delivery of miR-124 NPs we took advantage of the BBB breakdown caused by the PT stroke to facilitate the passage of miR-124 NPs into the brain parenchyma [41,42,56]. We also increase the amount of miR used for intravenous administration compared with the previous work [21] to guarantee the maximum load of miR into the NP formulation. In fact, zeta potential measures showed that at 1 h, at the concentration used in this study, miR NPs have a zeta potential near zero. After 24 h at 37°C both in static and flow conditions, miR NPs showed a decrease in the zeta potential in both conditions what may be explained by the adsorption of salts or oligonucleotides. However, it is also important to consider that after intravenous injection even the best formulations are only able to deliver up to 5% of their initial dose into the brain. As so, if around 0,1% of miR-124 NPs reached the brain parenchyma we would have around 4 pmol of miR-124 within the brain, 8-times more miR-124 than the amount locally administered in a Parkinson’s disease mouse model [21]. Immunofluorescence analysis of NPs and the increase of IL-6 levels in the post-ischemic brain strongly corroborate penetration of NPs and delivery of miR into brain cells despite we could not observe effects on neurogenesis in the SVZ, what may indicate low bioavailability. Differences in the route of administration (local vs systemic administration) might explain in part the lack of enhancement of SVZ neurogenesis. Nevertheless, improvement of our NP formulation is needed to increase efficacy of our treatment in systemic applications. Therefore, modifications that improve the blood circulation time, increase cargo protection (mainly against RNases), reduce opsonization, decrease peripheral accumulation and enhance delivery of miR into particular brain regions, such as addiction of polyethylene glycol, surfactant agents or even a targeting molecule, may increase bioavailability of the miR-124 [39].

## 5. Conclusions

In the present study, we showed that a single intravenous injection of miR-124 NPs immediately after PT does not affect neurological deficits 14 days following the insult. In addition, no effects have been observed on the number of neuronal progenitor cells in the subventricular zone and ischemic territory despite positive results from *in vitro* experiments. Further evaluation studies will be required to investigate bioavailability of miR-124 loaded nanoparticles in brain parenchyma, the immunogenic role of miR-124 NPs and its effect in different rodent stroke models.

## Supporting information

S1 Table Body weights and temperatures. Mean body weights and mean temperatures of sham- and PT-operated mice over the time course of the experimental procedure.
(DOCX)

## References

[1] Lagace DC. Does the endogenous neurogenic response alter behavioral recovery following stroke? Behav Brain Res. 2012;227: 426–432. doi: 10.1016/j.bbr.2011.08.045 21907736 10.1016/j.bbr.2011.08.045

[2] Lindvall O, Kokaia Z. Neurogenesis following Stroke Affecting the Adult Brain. Cold Spring Harb Perspect Biol. 2015;7: a019034 doi: 10.1101/cshperspect.a019034 26525150 10.1101/cshperspect.a019034 PMC4632663

[3] Marlier Q, Verteneuil S, Vandenbosch R, Malgrange B. Mechanisms and Functional Significance of Stroke-Induced Neurogenesis. Front Neurosci. 2015;9 doi: 10.3389/fnins.2015.00458 26696816 10.3389/fnins.2015.00458 PMC4672088

[4] Kuric E, Ruscher K. Dynamics of major histocompatibility complex class II-positive cells in the postischemic brain—influence of levodopa treatment. J Neuroinflammation. 2014;11: 145 doi: 10.1186/s12974-014-0145-z 25178113 10.1186/s12974-014-0145-z PMC4149192

[5] Lakhan SE, Kirchgessner A, Hofer M. Inflammatory mechanisms in ischemic stroke: therapeutic approaches. J Transl Med. 2009;7: 97 doi: 10.1186/1479-5876-7-97 19919699 10.1186/1479-5876-7-97 PMC2780998

[6] Simats A, Garcia-Berrocoso T, Montaner J. Natalizumab: a new therapy for acute ischemic stroke? Expert Rev Neurother. England; 2016;16: 1013–1021. doi: 10.1080/14737175.2016.1219252 27476862 10.1080/14737175.2016.1219252

[7] Bartel DP. MicroRNAs: genomics, biogenesis, mechanism, and function. Cell. 2004;116: 281–297. doi: 10.1016/S0092-8674(04)00045-5 14744438 10.1016/s0092-8674(04)00045-5

[8] Liu XS, Chopp M, Zhang RL, Tao T, Wang XL, Kassis H, et al MicroRNA Profiling in Subventricular Zone after Stroke : MiR-124a Regulates Proliferation of Neural Progenitor Cells through Notch Signaling Pathway. Baud O, editor. PLoS One. 2011;6: 1–11. doi: 10.1371/journal.pone.0023461 21887253 10.1371/journal.pone.0023461 PMC3162555

[9] Sun Y, Gui H, Li Q, Luo Z-M, Zheng M-J, Duan J-L, et al MicroRNA-124 protects neurons against apoptosis in cerebral ischemic stroke. CNS Neurosci Ther. 2013;19: 813–9. doi: 10.1111/cns.12142 23826665 10.1111/cns.12142 PMC6493643

[10] Laterza OF, Lim L, Garrett-Engele PW, Vlasakova K, Muniappa N, Tanaka WK, et al Plasma MicroRNAs as sensitive and specific biomarkers of tissue injury. Clin Chem. 2009;55: 1977–83. doi: 10.1373/clinchem.2009.131797 19745058 10.1373/clinchem.2009.131797

[11] Weng H, Shen C, Hirokawa G, Ji X, Takahashi R, Shimada K, et al Plasma miR-124 as a biomarker for cerebral infarction. Biomed Res. 2011;32: 135–141. doi: 10.2220/biomedres.32.135 21551949 10.2220/biomedres.32.135

[12] Liu Y, Zhang J, Han R, Liu H, Sun D, Liu X. Downregulation of serum brain specific microRNA is associated with inflammation and infarct volume in acute ischemic stroke. J Clin Neurosci. 2015;22: 291–5. doi: 10.1016/j.jocn.2014.05.042 25257664 10.1016/j.jocn.2014.05.042

[13] Rainer TH, Leung LY, Chan CPY, Leung YK, Abrigo JM, Wang D, et al Plasma miR-124-3p and miR-16 concentrations as prognostic markers in acute stroke. Clin Biochem. 2016;49: 663–668. doi: 10.1016/j.clinbiochem.2016.02.016 26968104 10.1016/j.clinbiochem.2016.02.016

[14] Doeppner TR, Doehring M, Bretschneider E, Zechariah A, Kaltwasser B, Müller B, et al MicroRNA-124 protects against focal cerebral ischemia via mechanisms involving Usp14-dependent REST degradation. Acta Neuropathol. 2013;126: 251–65. doi: 10.1007/s00401-013-1142-5 23754622 10.1007/s00401-013-1142-5

[15] Zhao C, Sun G, Li S, Lang M-F, Yang S, Li W, et al MicroRNA let-7b regulates neural stem cell proliferation and differentiation by targeting nuclear receptor TLX signaling. Proc Natl Acad Sci U S A. 2010;107: 1876–81. doi: 10.1073/pnas.0908750107 20133835 10.1073/pnas.0908750107 PMC2836616

[16] Hamzei Taj S, Kho W, Aswendt M, Collmann FM, Green C, Adamczak J, et al Dynamic Modulation of Microglia/Macrophage Polarization by miR-124 after Focal Cerebral Ischemia. J Neuroimmune Pharmacol. 2016;11: 733–748. doi: 10.1007/s11481-016-9700-y 27539642 10.1007/s11481-016-9700-y PMC5097787

[17] Hamzei Taj S, Kho W, Riou A, Wiedermann D, Hoehn M. MiRNA-124 induces neuroprotection and functional improvement after focal cerebral ischemia. Biomaterials. 2016;91: 151–65. doi: 10.1016/j.biomaterials.2016.03.025 27031810 10.1016/j.biomaterials.2016.03.025

[18] Zhu F, Liu J-L, Li J-P, Xiao F, Zhang Z-X, Zhang L. MicroRNA-124 (miR-124) Regulates Ku70 Expression and is Correlated with Neuronal Death Induced by Ischemia/Reperfusion. J Mol Neurosci. 2014;52: 148–155. doi: 10.1007/s12031-013-0155-9 24166354 10.1007/s12031-013-0155-9

[19] Liu X, Li F, Zhao S, Luo Y, Kang J, Zhao H, et al MicroRNA-124-Mediated Regulation of Inhibitory Member of Apoptosis-Stimulating Protein of p53 Family in Experimental Stroke. Stroke. 2013;44: 1973–1980. doi: 10.1161/STROKEAHA.111.000613 23696548 10.1161/STROKEAHA.111.000613

[20] Gomes RSM, Das Neves RP, Cochlin L, Lima A, Carvalho R, Korpisalo P, et al Efficient Pro-survival/angiogenic miRNA Delivery by an MRI-Detectable Nanomaterial. ACS Nano. 2013;7: 3362–72. doi: 10.1021/nn400171w 23451983 10.1021/nn400171w

[21] Saraiva C, Paiva J, Santos T, Ferreira L, Bernardino L. MicroRNA-124 loaded nanoparticles enhance brain repair in Parkinson’s disease. J Control Release. 2016;235: 291–305. doi: 10.1016/j.jconrel.2016.06.005 27269730 10.1016/j.jconrel.2016.06.005

[22] Agasse F, Bernardino L, Silva B, Ferreira R, Grade S, Malva JO. Response to histamine allows the functional identification of neuronal progenitors, neurons, astrocytes, and immature cells in subventricular zone cell cultures. Rejuvenation Res. 2008;11: 187–200. doi: 10.1089/rej.2007.0600 18279032 10.1089/rej.2007.0600

[23] Santos T, Ferreira R, Maia J, Agasse F, Xapelli S, Cortes L, et al Polymeric nanoparticles to control the differentiation of neural stem cells in the subventricular zone of the brain. ACS Nano. 2012;6: 10463–74. doi: 10.1021/nn304541h 23176155 10.1021/nn304541h

[24] Maia J, Santos T, Aday S, Agasse F, Cortes L, Malva JO, et al Controlling the neuronal differentiation of stem cells by the intracellular delivery of retinoic acid-loaded nanoparticles. ACS Nano. 2011;5: 97–106. doi: 10.1021/nn101724r 21171566 10.1021/nn101724r

[25] Bernardino L, Agasse F, Silva B, Ferreira R, Grade S, Malva JO. Tumor necrosis factor-alpha modulates survival, proliferation, and neuronal differentiation in neonatal subventricular zone cell cultures. Stem Cells. 2008;26: 2361–71. doi: 10.1634/stemcells.2007-0914 18583543 10.1634/stemcells.2007-0914

[26] Tennant KA, Adkins DL, Donlan NA, Asay AL, Thomas N, Kleim JA, et al The organization of the forelimb representation of the C57BL/6 mouse motor cortex as defined by intracortical microstimulation and cytoarchitecture. Cereb Cortex. United States; 2011;21: 865–876. doi: 10.1093/cercor/bhq159 20739477 10.1093/cercor/bhq159 PMC3059888

[27] Walter HL, van der Maten G, Antunes AR, Wieloch T, Ruscher K. Treatment with AMD3100 attenuates the microglial response and improves outcome after experimental stroke. J Neuroinflammation. BioMed Central; 2015;12: 24 doi: 10.1186/s12974-014-0232-1 25881123 10.1186/s12974-014-0232-1 PMC4329193

[28] Paxinos G, Franklin KBJ. The mouse brain in stereotaxic coordinates. 4th Revise San Diego, United States: Elsevier Science Publishing Co Inc; 2002.

[29] Ruscher K, Johannesson E, Brugiere E, Erickson A, Rickhag M, Wieloch T. Enriched environment reduces apolipoprotein E (ApoE) in reactive astrocytes and attenuates inflammation of the peri-infarct tissue after experimental stroke. J Cereb Blood Flow Metab. 2009;29: 1796–805. doi: 10.1038/jcbfm.2009.96 19623195 10.1038/jcbfm.2009.96

[30] Diederich K, Frauenknecht K, Minnerup J, Schneider BK, Schmidt A, Altach E, et al Citicoline Enhances Neuroregenerative Processes After Experimental Stroke in Rats. Stroke. 2012;43: 1931–1940. doi: 10.1161/STROKEAHA.112.654806 22581817 10.1161/STROKEAHA.112.654806

[31] Osman AM, Porritt MJ, Nilsson M, Kuhn HG. Long-term stimulation of neural progenitor cell migration after cortical ischemia in mice. Stroke. 2011;42: 3559–3565. doi: 10.1161/STROKEAHA.111.627802 21980195 10.1161/STROKEAHA.111.627802

[32] Freilich RW, Woodbury ME, Ikezu T. Integrated expression profiles of mRNA and miRNA in polarized primary murine microglia. PLoS One. 2013;8: e79416 doi: 10.1371/journal.pone.0079416 24244499 10.1371/journal.pone.0079416 PMC3823621

[33] Ruscher K, Kuric E, Liu Y, Walter HL, Issazadeh-Navikas S, Englund E, et al Inhibition of CXCL12 signaling attenuates the postischemic immune response and improves functional recovery after stroke. J Cereb Blood Flow Metab. 2013;33: 1225–34. doi: 10.1038/jcbfm.2013.71 23632969 10.1038/jcbfm.2013.71 PMC3734773

[34] Chapman KZ, Dale VQ, Dénes Á, Bennett G, Rothwell NJ, Allan SM, et al A Rapid and Transient Peripheral Inflammatory Response Precedes Brain Inflammation after Experimental Stroke. J Cereb Blood Flow Metab. 2009;29: 1764–1768. doi: 10.1038/jcbfm.2009.113 19654587 10.1038/jcbfm.2009.113

[35] Macas J, Nern C, Plate KH, Momma S. Increased generation of neuronal progenitors after ischemic injury in the aged adult human forebrain. J Neurosci. 2006;26: 13114–9. doi: 10.1523/JNEUROSCI.4667-06.2006 17167100 10.1523/JNEUROSCI.4667-06.2006 PMC6674966

[36] Lagos-Quintana M, Rauhut R, Yalcin A, Meyer J, Lendeckel W, Tuschl T. Identification of Tissue-Specific MicroRNAs from Mouse. Curr Biol. 2002;12: 735–739. doi: 10.1016/S0960-9822(02)00809-6 12007417 10.1016/s0960-9822(02)00809-6

[37] Cheng L-C, Pastrana E, Tavazoie M, Doetsch F. miR-124 regulates adult neurogenesis in the subventricular zone stem cell niche. Nat Neurosci. 2009;12: 399–408. doi: 10.1038/nn.2294 19287386 10.1038/nn.2294 PMC2766245

[38] Akerblom M, Sachdeva R, Barde I, Verp S, Gentner B, Trono D, et al MicroRNA-124 Is a Subventricular Zone Neuronal Fate Determinant. J Neurosci. 2012;32: 8879–8889. doi: 10.1523/JNEUROSCI.0558-12.2012 22745489 10.1523/JNEUROSCI.0558-12.2012 PMC4434222

[39] Saraiva C, Praça C, Ferreira R, Santos T, Ferreira L, Bernardino L. Nanoparticle-mediated brain drug delivery: Overcoming blood–brain barrier to treat neurodegenerative diseases. J Control Release. 2016;235: 34–47. doi: 10.1016/j.jconrel.2016.05.044 27208862 10.1016/j.jconrel.2016.05.044

[40] Jang J-W, Lee J-K, Hur H, Kim T-W, Joo S-P, Piao M-S. Rutin improves functional outcome via reducing the elevated matrix metalloproteinase-9 level in a photothrombotic focal ischemic model of rats. J Neurol Sci. 2014;339: 75–80. doi: 10.1016/j.jns.2014.01.024 24507948 10.1016/j.jns.2014.01.024

[41] Piao M-S, Lee J-K, Park C-S, Ryu H-S, Kim S-H, Kim H-S. Early activation of matrix metalloproteinase-9 is associated with blood–brain barrier disruption after photothrombotic cerebral ischemia in rats. Acta Neurochir (Wien). 2009;151: 1649–1653. doi: 10.1007/s00701-009-0431-1 19551335 10.1007/s00701-009-0431-1

[42] Nahirney PC, Reeson P, Brown CE. Ultrastructural analysis of blood–brain barrier breakdown in the peri-infarct zone in young adult and aged mice. J Cereb Blood Flow Metab. 2015; 0271678X15608396. doi: 10.1177/0271678X15608396 26661190 10.1177/0271678X15608396 PMC4759675

[43] Chen Y, Zhao H, Tan Z, Zhang C, Fu X. Bottleneck limitations for microRNA-based therapeutics from bench to the bedside. Pharmazie. 2015;70: 147–54. 91/ph.2015.4774 25980175

[44] van der Maten G, Henck V, Wieloch T, Ruscher K. CX3C chemokine receptor 1 deficiency modulates microglia morphology but does not affect lesion size and short-term deficits after experimental stroke. BMC Neurosci. 2017;18: 11 doi: 10.1186/s12868-016-0325-0 28061814 10.1186/s12868-016-0325-0 PMC5219711

[45] Anrather J, Iadecola C. Inflammation and Stroke: An Overview. Neurotherapeutics. United States; 2016;13: 661–670. doi: 10.1007/s13311-016-0483-x 27730544 10.1007/s13311-016-0483-x PMC5081118

[46] Veremeyko T, Siddiqui S, Sotnikov I, Yung A, Ponomarev ED. IL-4/IL-13-dependent and independent expression of miR-124 and its contribution to M2 phenotype of monocytic cells in normal conditions and during allergic inflammation. Block ML, editor. PLoS One. 2013;8: e81774 doi: 10.1371/journal.pone.0081774 24358127 10.1371/journal.pone.0081774 PMC3864800

[47] Ponomarev ED, Veremeyko T, Barteneva N, Krichevsky AM, Weiner HL. MicroRNA-124 promotes microglia quiescence and suppresses EAE by deactivating macrophages via the C/EBP-α–PU.1 pathway. Nat Med. 2011;17: 64–70. doi: 10.1038/nm.2266 21131957 10.1038/nm.2266 PMC3044940

[48] Ponomarev ED, Veremeyko T, Weiner HL. MicroRNAs are universal regulators of differentiation, activation, and polarization of microglia and macrophages in normal and diseased CNS. Glia. 2013;61: 91–103. doi: 10.1002/glia.22363 22653784 10.1002/glia.22363 PMC3434289

[49] Ponomarev ED, Maresz K, Tan Y, Dittel BN. CNS-derived interleukin-4 is essential for the regulation of autoimmune inflammation and induces a state of alternative activation in microglial cells. J Neurosci. 2007;27: 10714–21. doi: 10.1523/JNEUROSCI.1922-07.2007 17913905 10.1523/JNEUROSCI.1922-07.2007 PMC6672829

[50] Sun Y, Qin Z, Li Q, Wan J-J, Cheng M-H, Wang P-Y, et al MicroRNA-124 negatively regulates LPS-induced TNF-α production in mouse macrophages by decreasing protein stability. Acta Pharmacol Sin. 2016;37: 889–97. doi: 10.1038/aps.2016.16 27063215 10.1038/aps.2016.16 PMC4933752

[51] Qiu S, Feng Y, LeSage G, Zhang Y, Stuart C, He L, et al Chronic Morphine-Induced MicroRNA-124 Promotes Microglial Immunosuppression by Modulating P65 and TRAF6. J Immunol. 2015;194: 1021–1030. doi: 10.4049/jimmunol.1400106 25539811 10.4049/jimmunol.1400106 PMC4297711

[52] Sun Y, Li Q, Gui H, Xu D-P, Yang Y-L, Su D-F, et al MicroRNA-124 mediates the cholinergic anti-inflammatory action through inhibiting the production of pro-inflammatory cytokines. Cell Res. 2013;23: 1270–1283. doi: 10.1038/cr.2013.116 23979021 10.1038/cr.2013.116 PMC3817544

[53] Brennan GP, Dey D, Chen Y, Patterson KP, Magnetta EJ, Hall AM, et al Dual and Opposing Roles of MicroRNA-124 in Epilepsy Are Mediated through Inflammatory and NRSF-Dependent Gene Networks. Cell Rep. 2016;14: 2402–12. doi: 10.1016/j.celrep.2016.02.042 26947066 10.1016/j.celrep.2016.02.042 PMC4794429

[54] Zhao Y, Ma T, Chen W, Chen Y, Li M, Ren L, et al MicroRNA-124 Promotes Intestinal Inflammation by Targeting Aryl Hydrocarbon Receptor in Crohn’s Disease. J Crohn’s Colitis. Oxford University Press; 2016;10: 703–712. doi: 10.1093/ecco-jcc/jjw010 26802080 10.1093/ecco-jcc/jjw010

[55] Kimura A, Naka T, Nakahama T, Chinen I, Masuda K, Nohara K, et al Aryl hydrocarbon receptor in combination with Stat1 regulates LPS-induced inflammatory responses. J Exp Med. 2009;206: 2027–2035. doi: 10.1084/jem.20090560 19703987 10.1084/jem.20090560 PMC2737163

[56] Hoff EI, oude Egbrink MGA, Heijnen VVT, Steinbusch HWM, van Oostenbrugge RJ. In vivo visualization of vascular leakage in photochemically induced cortical infarction. J Neurosci Methods. 2005;141: 135–141. doi: 10.1016/j.jneumeth.2004.06.004 15585297 10.1016/j.jneumeth.2004.06.004

