# Supplementary material for: MicroRNA-124-loaded nanoparticles increase survival and neuronal differentiation of neural stem cells in vitro but do not contribute to stroke outcome in vivo
Source: PLoS One. 2018 Mar 1;13(3):e0193609. doi: 10.1371/journal.pone.0193609 (PMC5832317; doi:10.1371/journal.pone.0193609)
Supplement: S1 Table — Mean body weights and mean temperatures of sham- and PT-operated mice over the time course of the experimental procedure. (DOCX) [file pone.0193609.s001.docx]

**Supplementary material**

**MicroRNA-124-loaded nanoparticles increase survival and neuronal differentiation of neural stem cells *in vitro* but do not contribute to stroke outcome *in vivo***

Cláudia Saraiva^1,2,3^, Daniela Talhada^1,2^, Akhilesh Rai^4^, Raquel Ferreira^1^, Lino Ferreira^4,5^, Liliana Bernardino^1*^, Karsten Ruscher^2*^

^1^Health Sciences Research Centre, Faculty of Health Sciences, University of Beira Interior, 6200-506 Covilhã, Portugal; ^2^Laboratory for Experimental Brain Research, Division of Neurosurgery, Department of Clinical Sciences, Wallenberg Neuroscience Center, Lund University, BMC A13, 22184, Lund, Sweden; ^3^Departamento de Química, Faculdade de Ciências e Tecnologia da, Universidade Nova de Lisboa, 2829-516, Caparica, Portugal; ^4^CNC - Center for Neuroscience and Cell Biology, 3004-504 Coimbra, Portugal; ^5^Faculty of Medicine, University of Coimbra (IIIUC), 3030-789 Coimbra, Portugal.

*Corresponding authors: [libernardino@fcsaude.ubi.pt](mailto:libernardino@fcsaude.ubi.pt); [karsten.ruscher@med.lu.se](mailto:karsten.ruscher@med.lu.se).

S1 Table. Mean body weights and mean temperatures of sham- and PT-operated mice over the time course of the experimental procedure.

| **Treatment** | | **DAY 0** | | **DAY 2** | | **DAY 7** | | **DAY 14** | |
| --- | --- | --- | --- | --- | --- | --- | --- | --- | --- |
|  |  | **Body weight**  **(mean (g) ±SD)** | **Temperature (mean (ºC) ±SD)** | **Body weight**  **(mean (g) ±SD)** | **Temperature (mean (ºC) ±SD)** | **Body weight**  **(mean (g) ±SD)** | **Temperature (mean (ºC) ±SD)** | **Body weight**  **(mean (g) ±SD)** | **Temperature (mean (ºC) ±SD)** |
| **Sham** | Saline | 26.3 **±** 0.9 | 36.7 **±** 0.5 | 25.1 **±** 1.5 | 37.3 **±** 0.4 | - | - | - | - |
| **PT** | Saline | 25.9 **±** 1.6 | 36.7 **±** 0.7 | 24.5 **±** 1.3 | 37.4 **±** 0.6 | - | - | - | - |
| **PT** | Void NPs | 27.4 **±** 0.7 | 36.9 **±** 0.9 | 24.8 **±** 1.1 | 37.0 **±** 0.6 | - | - | - | - |
| **PT** | Scramble-miR NPs | 26.3 **±** 0.6 | 36.9 **±** 1.0 | 24.1 **±** 1.3 | 37.4 **±** 0.8 | - | - | - | - |
| **PT** | miR-124 NPs | 26.6 **±** 0.4 | 36.8 **±** 0.4 | 23.8 **±** 1.4 | 37.5 **±** 0.8 | - | - | - | - |
|  |  |  |  |  |  |  |  |  |  |
| **Sham** | Saline | 23.1 **±** 0.9 | 36.7 **±** 0.5 | 22.0 **±** 1.4 | 37.4 **±** 0.4 | 22.5 **±** 1.0 | 37.8 **±** 0.3 | 24.1 **±** 1.3 | 38.5 **±** 0.7 |
| **PT** | Saline | 22.8 **±** 1.4 | 36.9 **±** 0.5 | 21.3 **±** 1.4 | 37.6 **±** 0.5 | 22.0 **±** 1.2 | 37.9 **±** 0.5 | 23.4 **±** 1.1 | 38.0 **±** 0.5 |
| **PT** | Void NPs | 24.0 **±** 2.2 | 36.6 **±** 0.5 | 22.5 **±** 2.8 | 37.7 **±** 0.6 | 22.8 **±** 2.9 | 37.9 **±** 0.7 | 24.2 **±** 2.5 | 38.1 **±** 0.8 |
| **PT** | Scramble-miR NPs | 22.6 **±** 0.9 | 36.8 **±** 0.4 | 21.1 **±** 1.0 | 37.6 **±** 0.7 | 22.0 **±** 1.0 | 38.2 **±** 0.5 | 23.5 **±** 1.1 | 38.1 **±** 0.6 |
| **PT** | miR-124 NPs | 22.4 **±** 1.0 | 37.2 **±** 0.4 | 20.6 **±** 1.1 | 37.4 **±** 0.5 | 21.4 **±** 0.8 | 38.0 **±** 0.3 | 23.0 **±** 0.7 | 38.0 **±** 0.7 |
